# Adult Born Olfactory Bulb Dopaminergic Interneurons: Molecular Determinants and Experience-Dependent Plasticity

**DOI:** 10.3389/fnins.2016.00189

**Published:** 2016-05-06

**Authors:** Sara Bonzano, Serena Bovetti, Claudio Gendusa, Paolo Peretto, Silvia De Marchis

**Affiliations:** ^1^Department of Life Sciences and Systems Biology, University of TurinTorino, Italy; ^2^Neuroscience Institute Cavalieri Ottolenghi, University of TurinOrbassano, Italy; ^3^Department of Neuroscience and Brain Technologies, Istituto Italiano di TecnologiaGenova, Italy

**Keywords:** olfactory bulb, dopaminergic neurons, tyrosine hydroxylase, adult neurogenesis, COUP-TFI, juxtaglomerular neurons, odor enrichment, odor deprivation

## Abstract

The olfactory bulb (OB) is a highly plastic brain region involved in the early processing of olfactory information. A remarkably feature of the OB circuits in rodents is the constitutive integration of new neurons that takes place during adulthood. Newborn cells in the adult OB are mostly inhibitory interneurons belonging to chemically, morphologically and functionally heterogeneous types. Although there is general agreement that adult neurogenesis in the OB plays a key role in sensory information processing and olfaction-related plasticity, the contribution of each interneuron subtype to such functions is far to be elucidated. Here, we focus on the dopaminergic (DA) interneurons: we highlight recent findings about their morphological features and then describe the molecular factors required for the specification/differentiation and maintenance of the DA phenotype in adult born neurons. We also discuss dynamic changes of the DA interneuron population related to age, environmental stimuli and lesions, and their possible functional implications.

## Introduction

**Figure 1:**
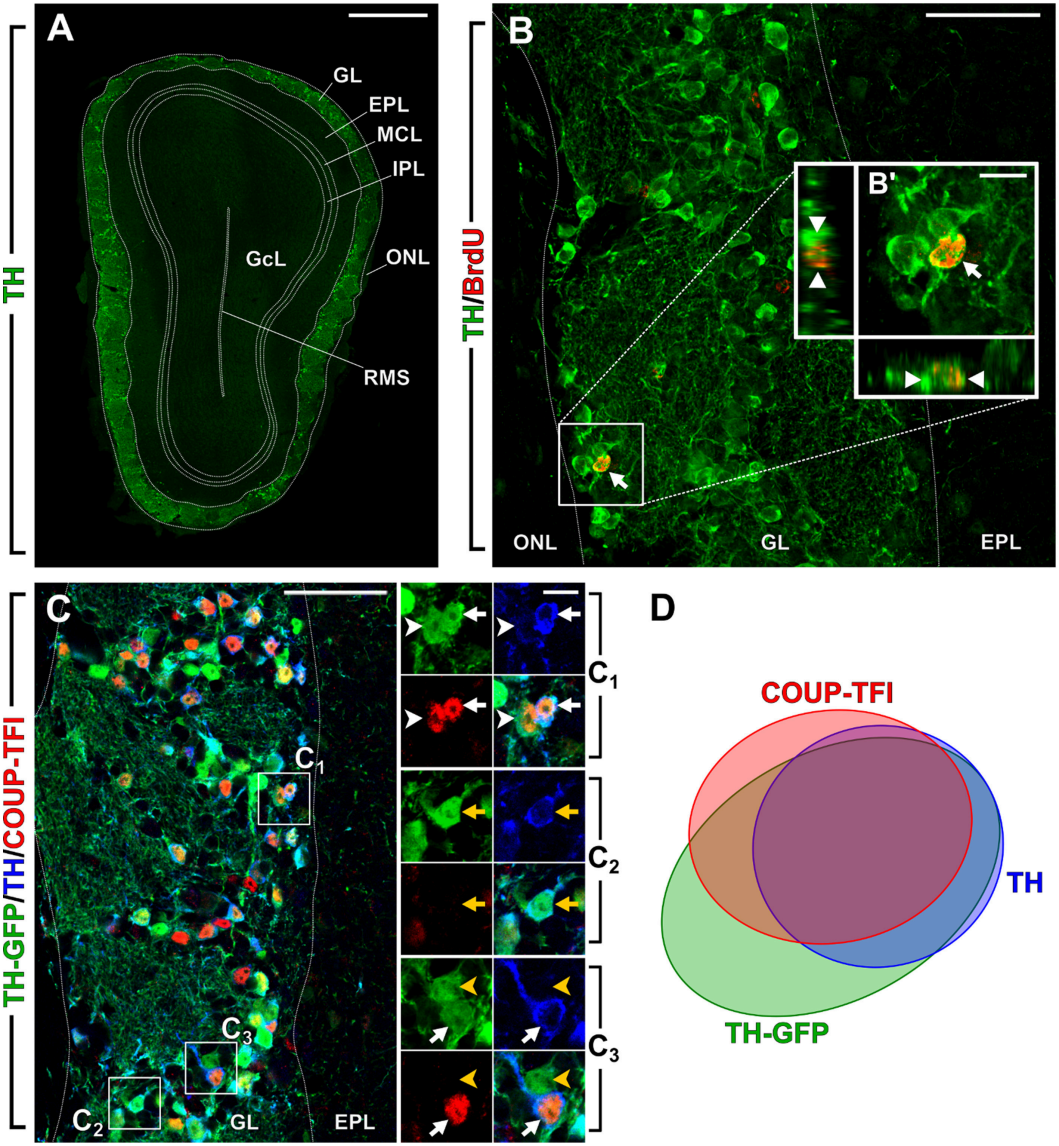
**Olfactory bulb dopaminergic interneurons**. New image from previously published experiments (Bovetti et al., 2013; Bonzano et al., 2014). **(A)** Photomicrograph showing a coronal section of the olfactory bulb (OB) in a 2-month-old wild-type mouse. DA cells immunopositive for TH (green) are mostly confined within the OB glomerular layer (GL). **(B)** BrdU-positive adult born DA cell (arrow) in a representative confocal image of the OB GL double-stained for BrdU (red) and TH (green) in a mouse that received BrdU at 2 months of age and analyzed 42 days after. B′ shows higher magnification and re-slicing of the BrdU/TH double positive cell. **(C)** Multiple labeling of the OB GL in a 2-month-old TH-GFP transgenic mouse line (Sawamoto et al., 2001). GFP (green) is expressed under the control of TH promoter; TH-immunopositive cells are shown in blue and COUP-TFI immunopositive nuclei in red. C1 shows higher magnification of a cell that is triple labeled for GFP/TH/COUP-TFI (white arrow) and a cell that is double labeled for GFP and COUP-TFI (white arrowhead). C2 shows higher magnification of a cell that is double labeled for GFP and TH (yellow arrow). C3 shows a cell that is triple labeled for GFP/TH/COUP-TFI (white arrow) and a cell that is GFP-positive only (yellow arrowhead). **(D)** Venn diagram showing the overlap of the labeling for TH-GFP, TH and COUP-TFI immunoreactivity based on our previously published data (Bovetti et al., 2013). A fraction of TH-GFP positive cells is negative for both TH and COUP-TFI. As previously reported these cells are likely immature DA neurons not expressing yet TH protein (Pignatelli et al., 2009). There is a high overlap between TH-GFP/TH/COUP-TFI labeling indicating that COUP-TFI expression is tightly associated with the DA phenotype. Scale bar in A = 500 μm. Scale bar in B and C = 50 μm. Scale bar in inset B′ = 10 μm. Scale bar in inset C1 = 10 μm and refers to C2 and C3. ONL, olfactory nerve layer; GL, glomerular layer; EPL, external plexiform layer; MCL, mitral cell layer; IPL, internal plexiform layer; GcL, granule cell layer; RMS, rostral migratory stream of the OB.

In mammals, dopaminergic (DA) neurons are classified in distinct neuronal cell groups (from A8 to A16) based on their substantial diversity (Björklund and Dunnett, 2007). DA neurons in the olfactory bulb (OB) belong to the A16 group and represent the major DA system in the forebrain (Cave and Baker, 2009). Olfactory DA cells are reliably identified by the expression of tyrosine hydroxylase (TH), the rate-limiting enzyme of catecholamine biosynthesis, since they represent the only catecholaminergic cell type found in the OB (Cave and Baker, 2009). TH-positive cells are mostly localized in the OB glomerular cell layer (GL; Figure 1A), where they account for nearly 10% of all juxtaglomerular cells (JGCs; Parrish-Aungst et al., 2007). TH-positive JGCs express glutamic acid decarboxylase (GAD), the rate-limiting enzyme for GABA biosynthesis, and co-release dopamine and GABA on their post-synaptic targets (Liu et al., 2013). Their electrophysiological properties have been extensively characterized (Pignatelli et al., 2005, 2009, 2013; Borin et al., 2014). TH-positive cells establish synaptic contacts with the afferent olfactory receptor neuron terminals and/or with external tufted cells and form extensive interglomerular connections (Kiyokage et al., 2010; Kosaka and Kosaka, 2011), participating to the early steps in odor information processing that occur in the input layer of the OB. Accordingly, a recent study demonstrated a key function for DA cells in implementing gain control and reducing correlation of odor representations in the main output neurons (i.e., mitral/tufted cells) (Banerjee et al., 2015). In line with a central role for DA cells in the encoding of odor stimuli, several studies support the impact of the DA system in fundamental features of odor-driven behaviors (Kruzich and Grandy, 2004; Pavlis et al., 2006; Tillerson et al., 2006; Wei et al., 2006; Serguera et al., 2008; Lazarini et al., 2014). Moreover, olfactory dysfunction is associated to pathological states affecting the DA system, such as in Parkinson disease (Doty, 2012). Although olfactory dysfunction in PD patients could also involve OB DA cells, recent data in rodents indicate this is mostly attributable to depletion in the DA nigro-olfactory projection system (Höglinger et al., 2015).

Olfactory DA neurons have attracted significant attention over the years, because they are involved in substantial activity-dependent plasticity, regulating the level of TH expression and dopamine release according to the sensory input (Nadi et al., 1981; Baker et al., 1983, 1984; Cummings et al., 1997). Moreover, DA cells are constantly generated throughout life and recent reports pointed to a specific integration of this juxtaglomerular cell population in the GL circuits in an activity-dependent manner (Sawada et al., 2011; Bonzano et al., 2014). Because of their continuous generation throughout life, DA cells are also regarded as a potential target to exploit adult neurogenesis for dopamine system repair in the brain (see Cave et al., 2014). Here we focused on emerging aspects related to DA cells heterogeneity, molecular determinants of adult born DA neurons, their plasticity and function in the OB.

## Olfactory DA interneurons belong to two main morphologically distinct cell populations

The view that DA cells in the OB consist of distinct types emerged many years ago from the immunohistochemical identification of two different categories of TH-positive neurons in the rat and mouse OB (Halász et al., 1981; Baker et al., 1983; McLean and Shipley, 1988). Based on their soma size and location, TH-positive cells were initially classified either as small periglomerular cells (PG, soma diameter about 5–10 μm), positioned in the GL and representing the large majority of olfactory DA cells, or large external tufted cells (ET, soma diameter about 10–15 μm), positioned mostly at the boundary between the GL and the external plexiform layer (EPL) and rarely found within the EPL. Interestingly, DA cells belonging to the larger type are born earlier during development than the smaller ones (McLean and Shipley, 1988), possibly from local OB progenitors in the E13.5 mouse embryo (Vergaño-Vera et al., 2006), before precursors from the main germinal niches for OB interneurons (i.e., LGE, pallium, and septum) start to populate the OB and differentiate into multiple interneuron subtypes (Bovetti et al., 2007; Alvarez-Buylla et al., 2008). Furthermore, Kosaka and Kosaka (2009) showed that adult subventricular zone (SVZ) progenitors do not contribute to the generation of the larger type of DA cells, indicating this population does not undergo the neuronal turnover typical of most GL interneurons, including small-medium sized DA cells (Bovetti et al., 2009).

Morphometric investigation of TH-positive neuronal projection in the GL has successively revealed that DA cells extend processes into multiple glomeruli (Kosaka and Kosaka, 2008; Kiyokage et al., 2010), suggesting that they should be more appropriately classified as short-axon (SA) cells instead of PG and ET cells, whose processes are mostly confined to one single glomerulus (Pinching and Powell, 1971; Kiyokage et al., 2010). Importantly, Kiyokage et al. (2010) described two distinct types of SA TH-positive cells, oligoglomerular and polyglomerular, based on their process extension and average number of contacted glomeruli. The vast majority of DA cells falls within the first category (i.e., oligoglomerular), having processes spanning a relatively short region of the GL and contacting in average nearly 6 glomeruli. Polyglomerular cells show more extensive projections, contact in average nearly 40 glomeruli and are likely to correspond to the large DA cells previously described to establish long-range interglomerular connections by Kosaka and Kosaka (2008). An additional feature that allows differentiating distinct types of olfactory DA cells is the presence/absence of an axon. Both *in vivo* (Kosaka and Kosaka, 2011) and *in vitro* (Chand et al., 2015) studies clearly indicated that larger DA cells possess an axon initial segment (AIS), reminiscent of an axonal process, while the other, smaller in size do not.

Overall, most evidences point to the existence of two main morphologically and possibly functionally separate populations of olfactory DA cells, of which only one (i.e., small/medium-sized DA neurons) undergoes continuous neurogenesis during adulthood (Figure 1B).

## Molecular determinants of the DA phenotype in the adult olfactory bulb

The generation of OB interneuron subtypes has been demonstrated to depend on a transcriptional code that is regulated in a spatio-temporal manner (Bovetti et al., 2007; Alvarez-Buylla et al., 2008). Distinct progenitor lineages differentially contribute to the generation of TH-positive cells during development or in adult mice. By means of genetic fate mapping Kohwi et al. demonstrated that while in neonates OB TH-positive cells only marginally (4%) derive from Emx1-expressing pallial progenitors, in adult age 42% of TH-positive OB interneurons are derived from this lineage (Kohwi et al., 2007). A prominent pallial origin of postnatal/adult DA interneurons is further supported by data obtained through adenoviral-mediated labeling of regionally restricted radial glial stem cells, showing that TH-positive neurons largely derive from progenitors located in the dorsal portion of the SVZ (Merkle et al., 2007). Several transcription factors (TFs), namely Pax6, Dlx2, Id2, Klf7, ER81, Sall3, Nurr1, and Meis2 (Saino-Saito et al., 2004, 2007; Hack et al., 2005; Kohwi et al., 2005; Brill et al., 2008; Havrda et al., 2008; Cave et al., 2010; Caiazzo et al., 2011; Heng et al., 2012; Agoston et al., 2014; Vergaño-Vera et al., 2015) have been shown to be required for proper differentiation of olfactory DA neurons. Here we will limit the review to those TFs whose function in the control of olfactory DA fate has been directly demonstrated in adult born neurons (Table 1). Among these, Pax6 and Dlx2 play a major role (Hack et al., 2005; Kohwi et al., 2005; Brill et al., 2008; de Chevigny et al., 2012). The use of retroviral-mediated overexpression of Dlx2 in neuronal precursors along the rostral migratory stream (RMS) provided data supporting a cell-autonomous role for this TF in promoting specification of adult born neurons toward DA fate (Brill et al., 2008). Similar results, implying increased generation of DA interneurons, were previously described by over-expressing the TF Pax6 in adult neuronal precursors migrating along the RMS (Hack et al., 2005). In addition, by increasing Pax6 protein level in the lateral wall, where normally Pax6 protein is absent due to post-transcriptional inhibition by mir-7a, the acquisition of the DA phenotype in the OB is favored (de Chevigny et al., 2012). Moreover, the effect of Dlx2 overexpression is totally abrogated in the absence of Pax6 and functional direct interaction/cooperation between Dlx2 and Pax6 is needed to instruct DA fate in adult mice (Brill et al., 2008). A critical co-factor for Pax6 and Dxl2 function in exploiting DA fate commitment in adult born OB interneurons has been recently identified in Meis2, a member of the three amino acid loop extension class of atypical homeodomain-containing transcription factors (Agoston et al., 2014). Meis2, together with Pax6 and Dlx2, is needed to determine the differentiation toward a DA phenotype over the CR one by directly interacting with TH regulatory sequences (Agoston et al., 2014). Besides the instructive role for Pax6 in DA fate commitment in OB interneuron precursors, Pax6 is also critically involved in OB DA cell maintenance. Indeed, by conditionally deleting Pax6 in mature DA cells Ninkovic et al. (2010) identified Pax6 as a positive controller of mature DA cell survival through the positive regulation of crystallin αA in the adult OB.

**Table 1:** **Transcription factors involved in the control of adult born olfactory DA fate specification and maintenance**.

**TF**	**% of JGC type among TF+ cells**	**% of TF+ among TH+ cells**	**Experimental strategies**		**Phenotype**	**References**
			**Type of approach**	**Cellular/area targets**		
Pax6	TH 78% CR 2% CB 10%	95%	- RV stereotaxic injection(overexpression)	RMS/SVZ (adult born)	Increase in adult born TH+ cells (14/21/90 dpi)	Hack et al., 2005
			-RV stereotaxic injection (loss of function)	RMS (adult born)	Decrease in adult born TH+ cells (21 dpi)	Hack et al., 2005
			-Transplantation of dLGE Pax6-deficient E16.5 progenitors	Recipient: wt adult SVZ	Decrease in TH^+^ cells among grafted cells (40 dpt)	Kohwi et al., 2005
			-Conditional KO (Dat-Cre^*^ Pax6fl/fl)	Unspecific to adulthood (mature DA cells)	Decrease in TH^+^ cells; Decrease in DA cell survival	Ninkovic et al., 2010
			-Pax6+/Sey^*Dey*^	Unspecific to adulthood	Decrease in adult born TH+ cells; decrease in DA cell survival (15–60 dpBrdU)	Curto et al., 2014
			-Pax6-ORF-GFP plasmid electroporation - Ectopic Pax6 expression in lateral SVZ	Postnatal lateral SVZ	Increase in TH+ cells (15 dpe)	de Chevigny et al., 2012
Dlx2	TH unknown CR none CB unknown	Virtually all	-RV stereotaxic injection (overexpression)	RMS (adult born)	Increase in TH+ cells paralleled by decreased CR+ cells (21–56dpi)	Brill et al., 2008
Meis2	Unknown exactly	89%	−	−	−	Allen et al., 2007
		94%	-RV stereotaxic injection (loss of function)	RMS (adult born)	Loss of adult born TH+ cells (21/60 dpi)	Agoston et al., 2014
COUP-TFI	TH 70% CR 1% CB 2%	80%	-LV stereotaxic injection (loss of function)	RMS (adult born)	Decrease in adult born TH+ cells (60 dpi); no changes at 30 dpi	Bovetti et al., 2013
			Conditional KO (Emx1-Cre^*^COUP-TFIfl/fl)	Unspecific to adulthood	Decrease in TH+ cell population; decrease in the % of TH+ on BrdU adult born OB INs (42dpBrdU); no changes in DA cell survival	Bovetti et al., 2013; Zhou et al., 2015

*TH, tyrosine hydroxylase; TF, transcription factor; CR, calretinin; CB, calbindin; dpi, day(s) post injection; dpBrdU, day(s) post BrdU injection(s); dpe, day(s) post electroporation; dpt, day(s) post transplantation; RV, Retroviral vector; LV, Lentiviral vector; RMS, Rostral Migratory Stream; SVZ, Sub Ventricular Zone; dLGE, dorsal Lateral Ganglionic Eminence*.

Recently, we have identified a distinct, yet central, role in the maintenance of the DA phenotype of adult born OB interneurons for the orphan nuclear receptor COUP-TFI (Bovetti et al., 2013). Among juxtaglomerular interneurons in the adult mouse OB, COUP-TFI expression is exclusively found in DA cells, with nearly 80% of mature TH-positive cells (Figure 1C,D) and 90% of Pax6-positive cells double positive for COUP-TFI. Interestingly, the expression of COUP-TFI is mostly confined to DA cells generated during late postnatal/adult life, and is regulated by the sensory input. Indeed, odor deprivation through naris occlusion induces COUP-TFI down-regulation jointly with TH down-regulation in olfactory DA cells. Moreover, we observed a net impairment in TH expression in fully mature cells following ablation of COUP-TFI function in either a) DA interneuron progenitors by means of conditional COUP-TFI deletion in the Emx1-lineage or b) post-mitotic adult born neurons by lentiviral-mediated approach *in vivo* (Bovetti et al., 2013). These findings strongly indicate a role for COUP-TFI in TH expression regulation, as also recently confirmed by another study (Zhou et al., 2015). Importantly, COUP-TFI ablation on DA cells does not affect the acquisition and maturation of the DA phenotype, but impairs immediate early gene expression (i.e., egr-1; Bovetti et al., 2013). Overall, these data, together with the apparent lack of consensus binding sites for COUP-TFI on the TH promoter, strongly indicate that COUP-TFI regulates TH expression in OB cells through an activity-dependent mechanism involving immediate early gene induction and strongly argue for a maintenance rather than establishment function of COUP-TFI in the DA commitment. Thus, besides the role of TFs such as Pax6, Meis2, and Dlx2 that are directly involved in OB DA fate determination within adult SVZ neural stem cell/precursors, COUP-TFI must be part of a distinct transcription factor program that is central for the maintenance of the DA cell identity over time.

## Experience-dependent plasticity of olfactory DA neurons: a dual mechanism involving TH-expression regulation and adult neurogenesis

Several lines of evidence support the notion that olfactory DA neurons are unique among OB neurons, being particularly susceptible to sensory stimuli. A first level of experience-dependent plasticity of DA cells consists in the regulation of TH expression and consequently dopamine production/release, according to the sensory input. Indeed, TH expression in DA cells is strongly and reversibly down-regulated in animals subjected to odor deprivation by either chemical or surgical sensory deafferentation of the OB (Nadi et al., 1981; Kawano and Margolis, 1982; Baker et al., 1983), or naris occlusion (Baker et al., 1993). This effect does not seem to be restricted to a specific DA cell population (see above; Baker et al., 1983) and applies to both pre-existing and adult generated neurons (Bovetti et al., 2009; Bastien-Dionne et al., 2010). In parallel to TH down-regulation, odor deprivation also induces down-regulation of GAD67, which is selectively co-expressed by DA cells, but not of GAD65, which is mainly expressed by other juxtaglomerular cell types (Parrish-Aungst et al., 2011). Although the view that olfactory DA neurons are exposed to modulation of their transmitter phenotype by the olfactory input has long been recognized (Baker et al., 1983, 1984; McLean and Shipley, 1988), the molecular mechanisms underlying this phenomenon are just beginning to emerge. A direct link among the expression of immediate early genes, increased neuronal activity and TH expression in the GL has been previously hypothesized (Jin et al., 1996). As reported above, we recently identified a role for DA cell responsiveness to sensory stimuli for COUP-TFI, whose depletion in adult generated DA cells induces both reduced immediate early gene and TH expression (Bovetti et al., 2013). Moreover, recent studies highlighted the involvement of epigenetic regulatory mechanisms in the activity-dependent modulation of the neurotransmitter phenotype in OB interneurons (Banerjee et al., 2013).

Besides TH expression regulation, sensory activity can significantly impact on the composition of the DA population through modulation of adult neurogenesis. Indeed, manipulation of the sensory input by either odor deprivation or enrichment elicits, respectively, decreased or increased survival of adult generated juxtaglomerular interneurons (Bovetti et al., 2009), as previously demonstrated for granule cells (Rochefort et al., 2002; Mandairon et al., 2006). Increasing evidence points to DA cells as a selective cellular target for sensory-dependent modulation of adult neurogenesis in the GL of the OB. Using a paradigm of naris occlusion in adult mice, Sawada et al. (2011) found that among different neurochemical types of juxtaglomerular cells, TH-positive DA cells were the only one to show increased apoptosis. Interestingly, mice in which the naris was reopened showed increased integration of new DA cells after a 4 weeks recovery phase that compensate for the selective loss of DA cells due to previous deprivation. A restorative role of adult neurogenesis has been also demonstrated in another experimental paradigm in which a selective DA neuronal loss, induced by local treatment with 6-hydroxydopamine (6-OHDA) in the dorsal OB, was followed by a full recovery of DA cells (Lazarini et al., 2014). Regulation of DA cell generation in adulthood is not limited to restorative conditions but occurs also in basal physiological condition and in response to sensory enrichment. Interestingly, a net and selective increase in the glomerular DA population with age has been reported in a long term two-photon imaging study *in vivo* (Adam and Mizrahi, 2011). Although the meaning of these age-dependent changes in the DA population is unknown, these data reinforce the idea that a certain plasticity of the DA population is required for OB circuit functions. In a recent study from our group (Bonzano et al., 2014), a paradigm of prolonged (2 months) olfactory enrichment with different aromatic fragrances, which has been previously shown to affect OB neurogenesis (Rochefort et al., 2002; Bovetti et al., 2009) and olfactory memory (Rochefort et al., 2002), resulted in a selective increase in the TH-positive DA population, due to increased neurogenesis, without changes in calretinin (CR)- and calbindin (CB)-positive neurons (Bonzano et al., 2014). These results further support that adult neurogenesis does not reflect a simple turnover of the whole GL interneuron population, but it can finely modulate specific OB neuron subpopulations (i.e., DA cells) with particular functions in odor processing.

## Conclusion and future perspective

In the adult OB, DA cells are unique in term of their plasticity in response to sensory inputs. Although their involvement in mechanisms underlying the adaptation of the olfactory system to changes in sensory experience is well established, many aspects remain still unknown. The heterogeneity of DA cell population in term of morphology, connections (Kosaka and Kosaka, 2008; Kiyokage et al., 2010; Chand et al., 2015), origin (McLean and Shipley, 1988; Vergaño-Vera et al., 2006; De Marchis et al., 2007; Kohwi et al., 2007; Merkle et al., 2007) and renewal (Kosaka and Kosaka, 2009) further complicate the understanding of DA cell role in odor coding, processing and plasticity. New molecular and optical approaches able to selectively target adult born DA interneurons will hopefully bring new insights in unraveling their role in olfactory physiology. The precise *in vivo* readout of cell activity now possible exploiting the last generation of calcium and voltage indicators (Akemann et al., 2010; Chen et al., 2013; Gong et al., 2015), combined with the capability to selectively manipulate cell activity through optogenetic and chemogenetic tools (Boyden et al., 2005; Deisseroth et al., 2006; Liu et al., 2013; Sternson and Roth, 2014), are the straightforward direction toward the complete dissection of glomerular network function and adult born DA cell role in activity-dependent plasticity. Nonetheless, new molecular and genetic tools may contribute to further clarify and reach a final consensus on olfactory DA cell classification.

## Author contributions

All authors listed, have made substantial, direct, and intellectual contribution to the work, and approved its final version for publication.

## Funding

This work was supported by the University of Turin (ex 60% 2014–2015 to SDM).

### Conflict of interest statement

The authors declare that the research was conducted in the absence of any commercial or financial relationships that could be construed as a potential conflict of interest.
